# Azathioprine-induced toxoplasma gondii infection in a patient with Crohn's disease with NUDT15 variation: A case report

**DOI:** 10.1097/MD.0000000000025781

**Published:** 2021-05-07

**Authors:** Yanan Wu, Yuyong Tan, Dalian Ou, Xuehong Wang, Yongjun Wang

**Affiliations:** a Department of Gastroenterology, The Second Xiangya Hospital; b Research Center of Digestive Disease, Central South University, Changsha, Hunan 410011, China.

**Keywords:** azathioprine, Crohn's disease, NUDT15, toxoplasma gondii infection

## Abstract

**Introduction:**

Azathioprine (AZA) has been widely used for the treatment of various immune-related diseases and has become a mainstay in the treatment of inflammatory bowel disease. However, patients with genetic mutations may experience severe adverse events when treated with azathioprine. Most of the previous literature focused on the TPMP gene-related adverse reactions, herein, we report a case of Crohn's disease patient with nucleoside diphosphate-linked moiety X motif 15 gene (NUDT15) variation and wild-type TPMP gene who developed toxoplasma gondii infection after azathioprine treatment.

**Patient concerns:**

A 56-year-old Crohn's disease patient developed toxoplasma gondii infection within 2 months after the administration of azathioprine; however, he had no relevant high-risk factors.

**Diagnosis:**

Subsequent genetic testing revealed that the patient was heterozygous for NUDT15. Therefore, it was reasonable to consider that the patient's genetic mutation resulted in reduced tolerance to azathioprine, leading to low immunity and eventually toxoplasma infection.

**Interventions:**

AZA was then discontinued; after anti-infection, antipyretic and other supportive treatments were administered, the patient's condition gradually improved.

**Outcomes:**

The patient was followed up at 1, 3, and 6 months after discharge; fortunately, he was in good health.

**Conclusion:**

We report a case of Crohn's disease in a patient who developed severe pneumonia caused by toxoplasma gondii infection due to the administration of AZA, with normal TPMP gene but NUDT15 gene mutation. This indicates that NUDT15 variation may contribute to severe adverse events in patients treated with azathioprine, and we suggest that NUDT15 genotype be detected before the use of azathioprine in order to provide personalized therapy and reduce side effects.

## 1 Introduction

Azathioprine (AZA), a prodrug of 6-mercaptopurine (6-MP), has been widely used as an immunomodulator in many diseases, such as acute lymphoblastic leukemia, inflammatory bowel disease (IBD), rheumatoid arthritis, autoimmune hepatitis, and prevention of rejection after organ transplant,^[1–3]^ and has become a mainstay in the treatment of IBD because of its effectiveness and relatively low prices. However, 15% to 30% of patients discontinue therapy because of AZA-associated adverse drug reactions.^[4]^ Generally, and most side effects can be divided into 2 groups based on whether they are dose dependent. The clinical manifestations of non-dose-dependent adverse reactions such as nausea, vomiting, hypohepatia, and pancreatitis. Dose-dependent side effects manifest in hematopoietic damage, mainly in bone marrow suppression,^[5–7]^ so if a lower dose of AZA is administered or discontinuing the therapy, the adverse effects will fade away. Thiopurine-S-methyltransferase (TPMT) is a key enzyme in AZA metabolism and is associated with AZA-induced leukopenia.^[8]^ Patients carrying nonfunctional TPMP alleles require treatment cessation or dose adjustment. Therefore, the Clinical Pharmacogenetics Implementation Consortium suggests that Asians should be tested for the TPMT∗3C and TPMT∗3A genotypes before starting treatment with AZA.^[9]^ Nevertheless, the frequency of TPMP variation is rare in Chinese patients, approximately 0.9%,^[10]^ which limits the clinical usefulness of TPMP genotype detection. In recent years, some studies have indicated that the nucleoside diphosphate-linked moiety X motif 15 gene (NUDT15) is a novel predictor of thiopurine-induced leukopenia. Yang first identified the role of NUDT15-rs116855232 in Korean subjects with Crohn's disease treated with thiopurines in 2014^[11]^; in this paper, we present a case of a Crohn's disease (CD) patient receiving oral AZA with mutant (CT allele) NUDT15-rs116855232 and normal TPMP activity, who developed severe pneumonia caused by toxoplasma gondii infection.

## 2 Case presentation

In March 2018, a 56-year-old Chinese patient underwent colonoscopy due to changes in stool characteristics, and postoperative pathology indicated that the lesion was consistent with CD. The patient was previously free of infectious diseases or other serious diseases. The patient was initially treated with mesalazine (1 g per session, twice daily) and was discharged after her symptoms improved. One month later, the patient visited the hospital again because of abdominal pain. Gastroscopy and colonoscopy were performed on the patient, and the pathological report indicated that the patient had a relatively serious inflammatory stage of CD. Considering the poor control of CD, AZA and methylprednisolone were used instead. The initial dose of AZA was about 1.41 mg/kg/day without gene detection. Routine blood tests did not reveal any obvious abnormalities. Unfortunately, the patient developed fever, cough, and sputum after taking AZA only 2 months later. A CT scan of the lung in another hospital suggested bilateral interstitial lung lesions complicated by infection. Parasite detection revealed that the antibody against toxoplasma gondii was 1:128 ++, and other etiological evidence of infection was not found. Toxoplasma infection was diagnosed after a complete examination. Genetic testing revealed that the patient's TPMP gene was wild type, but had a NUDT15 gene heterozygous mutation. AZA was then discontinued, after anti-infection, antipyretic, and other supportive treatments were administered, the patient's condition gradually improved. Detailed patient information is provided in Table 1. The lymphocyte subsets of the patients also indicated a weakened immune system (Table 2). The patient was followed up at 1, 3, and 6 months after discharge; fortunately, he was in good health. We obtained informed consent from the patients for publication of this case report. Combined with the patient's laboratory examination and medical history, it is reasonable to believe that the patient's tolerance to AZA was reduced due to mutations in the NUDT15 gene, and the patient's immune function was severely suppressed, leading to severe pneumonia caused by toxoplasma infection.

**Table 1:** Characteristics of the patient.

Characteristics	
Age, yrs	56
Gender	Male
Profession	Teacher
Diagnosis	Crohn's disease
Weight, kg	71
AZA dose, mg/kg/d	1.41
Date at onset of AZA treatment, mo/d/yr	05/22/2018
Initial time of adverse reactions, mo/d/yr	07/20/2018
NUDT15-rs116855232	CT (heterozygote) (poor metabolizer)
TPMP-rs1142345	AA (wild type) (normal metabolizer)

AZA = azathioprine.

**Table 2:** Examination results of the patient.

Items	Outcome	Reference value
TOX-IgM	+	−
TOX-IgG	+	−
Toxoplasma gondii IHA	≥1:128 (++)	−
%CD3+	69	56–86%
#CD3+	212	723–2733/μL
%CD3 + CD4+	43	33–58%
#CD3 + CD4+	134	404–1612/μL
%CD3 − CD19+	18	5–22%
#CD3 − CD19+	47	80–616/μL

CD = Crohn's disease, IHA = indirect hemagglutination assay.

## 3 Discussion

AZA is a prodrug of 6-MP that metabolizes to its active form through a complex metabolic pathway. Briefly, AZA converts to 6-MP through glutathione S-transferase, which then undergoes 3 major metabolic pathways by the enzymes hypoxanthine phosphoribosyl transferase, TPMT, and xanthine oxidase, respectively. As the main active metabolite responsible for therapeutic efficacy, 6-thioguanine nucleotide (6-TGN) is converted by 6-MP through multiple enzymes, including hypoxanthine phosphoribosyltransferase, TPMP, inosine monophosphate dehydrogenase, and GMPS. 6-Thiouric acid and 6-methylmercaptopurine are the metabolites of the remaining 2 pathways that are inactive.^[12,13]^ Generally, 6-TGN reaches its steady-state after 4 to 8 weeks of the initiation of AZA therapy.^[14]^ The therapeutic range has been defined as a steady-state 6-TGN concentration between 235 and 450 pmol/8 × 10^8^ red blood cells (RBCs).^[12,15]^ Low doses of 6-TGN may lead to decreased efficacy whereas high 6-TGN levels could increase the risk of myelosuppression,^[16]^ which could induce life-threatening adverse effects. The enzyme activity of patients with nonfunctional genes decreases, leading to the accumulation of 6-TGN, which then results in adverse reactions.^[12,16,17]^ TPMP activity is highly variable among patients due to genetic polymorphism in the TPMP gene. Therefore, previous studies suggested that TPMP gene detection before AZA was used.^[8,9]^ There are also many factors that can affect enzyme activity, accounting for genotype and phenotype inconsistencies, such as recent blood transfusion,^[18]^ age, and gender.^[19,20]^ Compared with the frequency of TPMT mutations in Caucasians (∼10%), the frequency is rare in Asians (1–3%), especially in China (0.9%), which is contradictory to the higher occurrence of thiopurine-induced myelosuppression in Asians (30–40%).^[10,21,22]^ Thus, as a novel predictor of AZA-associated side effects, NUDT15 was first identified by Yang et al in 2014.^[11]^ In this study, NUDT15 risk allele is more common in Asians than in individuals of European descent, with mutant allele frequencies of 13% in Chinese, 10.4% in Koreans, 7% in Japanese, and 2% in an admixed American population. Ailing^[23]^ reported the first case of a 40-year-old Chinese primary biliary cirrhosis and autoimmune hepatitis overlap syndrome with AZA-induced severe toxicity with no clinically significant TPMT variant but with the NUDT15 c. 415C > T allele, further illustrating that NUDT15 might be another factor in predicting AZA-associated adverse events.

As mentioned earlier, TGN is the main active metabolite of AZA. TGN is a compound that consists of 6-thioguanine-monophosphate, 6-thioguanine-diphosphate, and 6-thioguanine-diphosphate-triphosphate (6-TGTP).^[13]^ After 6-TGTP transformed into 6-deoxythioguanosine-triphosphate (6-TdGTP), 6-TGTP and 6-TdGTP can lead to apoptosis through incorporation into RNA or DNA to trigger futile mismatch repair.^[24–26]^ NUDT15 is a 164-amino-acid protein belonging to the nudix hydrolase enzyme family and its role is to dephosphorylate 6-TGTP and 6-TdGTP, preventing them from infiltrating DNA.^[27,28]^ Therefore, the cytotoxic effects of AZA are reduced by removing the oxidatively damaged nucleotides from cells. Unfortunately, the specific pathogenesis mechanism of NUDT15 variation in human subjects is unclear. Yang et al^[11]^ reported that the sensitivity and specificity of variant NUDT15 for predicting thiopurine-induced early leukopenia was 89.4% and 93.2%, respectively. It also revealed that the risk of developing thiopurine-induced early leukopenia among individuals with 1 copy of the NUDT15 risk allele was significantly increased by 88-fold compared to non-carriers. In recent years, severe adverse reactions have been reported in patients with mutations in the NUDT15 gene and wild TPMP gene after treatment with AZA, with leukopenia and alopecia being the most common,^[23,29–35]^ indicating the necessity of determining NUDT15 activity status prior to treatment with AZA. In addition, NUDT15_rs116855232 might have a higher diagnostic accuracy than NUDT15_rs554405994 and NUDT15_rs186364861.^[36]^ Relling^[37]^ recommended a dosing strategy of AZA by NUDT15 phenotype. Starting with a normal starting dose (e.g., 2–3 mg/kg/day) in patients with normal NUDT15 activity and dose adjustment based on disease-specific guidelines. For patients with deficiency of enzyme activities, AZA doses were adjusted based on the degree of myelosuppression and disease-specific guidelines. More specifically, start 30% to 80% of the normal dose (for example, 0.6–2.4 mg/kg/day) for NUDT15 intermediate metabolizer, and drastically reduced normal daily doses (reduced daily dose by 10-fold) or an alternative immunosuppressant therapy for NUDT15 poor metabolizer, including switching to 6-MP or thioguanine, desensitization, subdivide the total ideal thiopurine dose into 2 smaller daily doses.^[12]^ Furthermore, Wang et al^[20]^ have showed that the combined use of corticosteroids may decrease the risk of developing AZA-induced leukopenia. Toxoplasma gondii is one of the most widely known zoonotic pathogens.^[38]^ People at high risk of infection of toxoplasma gondii are pregnant women, fetuses, occupational people who have long-term contact with livestock or raw meat, and immunocompromised people, such as AIDS patients.^[39,40]^ Immunosuppressive therapy has been shown to affect parasite load times in toxoplasma infected mice.^[41]^ In recent years, most cases of toxoplasma infection have been reported in organ transplant patients,^[42–44]^ while reports of IBD patients infected with hormones and immunosuppressive agents are very rare. According to the key words “Inflammatory bowel disease,” “azathioprine,” and “Toxoplasma,” only 1 relevant article was found in the database.^[45]^ The patient in our study is not at high risk for toxoplasma infection on account of no previous history of long-term hormone and immunosuppressive medication or leukopenia, more importantly, he used a relatively conservative starting dose (1.41 mg/kg/day). It is reasonable to think that the patient's genetic mutation leads to severe humoral immunosuppression after the administration of AZA, resulting in severe pneumonia caused by toxoplasma infection. The patient in this case did not suffer leukopenia, but a severe infection occurred, suggesting that genotype testing should be performed even if the patient's white blood cells were normal. A worldwide survey showed that thiopurine testing is relatively underutilized by physicians,^[46]^ but genetic testing has been proven to be cost effective. Given the rate of hospitalization, the cost of medication and treatment caused by AZA cytotoxicity, the high prices of genetic testing are worthwhile.^[5]^

It is worth noting that although wild-type patients have a relatively low risk of adverse reactions, they do not represent absolute safety. This highlights the importance of monitoring and preventing adverse reactions after AZA treatment. Thiopurine-induced adverse drug reactions are multifactorial events, and in addition to focusing on the genotype of the patient, the age and gender of the patients, concomitant use of medications, baseline WBC count, and smoking status^[16,47,48]^ should be noted.

## 4 Conclusion

We report a case of severe pneumonia caused by toxoplasma gondii infection due to the administration of AZA in a patient with Crohn's disease, with a normal TPMP gene but a NUDT15 gene mutation. This study further verified the view of previous studies that NUDT15 gene mutations could cause serious adverse reactions. Opportunistic infections should also be considered. In conclusion, genetic testing before the use of AZA is necessary to improve efficacy and reduce adverse reactions.

## Author contributions

Yanan Wu: Study concept and design, data extraction and statistical analysis, interpretation of the data, manuscript writing, critical revision of the article for important intellectual content, and approval of the final article for submission.

Yuyong Tan: Interpretation of the data, critical revision of the article for important intellectual content, manuscript writing, and approval of the final article for submission.

Dalian Ou: Interpretation of the data, critical revision of the article for important intellectual content, and approval of the final article for submission.

Xuehong Wang: Interpretation of the data, design of methodology, critical revision of the article for important intellectual content, and approval of the final article for submission.

Yongjun Wang: Ideas, formulation of overarching research goals, critical revision of the article for important intellectual content, and approval of the final article for submission.

**Conceptualization:** Yanan Wu, Yongjun Wang.

**Data curation:** Yanan Wu, Yuyong Tan, Dalian Ou, Xuehong Wang.

**Formal analysis:** Yuyong Tan.

**Investigation:** Yanan Wu, Yongjun Wang.

**Methodology:** Xuehong Wang.

**Resources:** Yongjun Wang.

**Supervision:** Yanan Wu, Yuyong Tan, Dalian Ou, Xuehong Wang.

**Validation:** Yanan Wu, Yuyong Tan, Dalian Ou, Xuehong Wang, Yongjun Wang.

**Writing – original draft:** Yanan Wu.

**Writing – review & editing:** Yanan Wu, Yuyong Tan, Dalian Ou, Xuehong Wang, Yongjun Wang.
